# Vaccinia virus-mediated cancer immunotherapy: cancer vaccines and oncolytics

**DOI:** 10.1186/s40425-018-0495-7

**Published:** 2019-01-09

**Authors:** Zong Sheng Guo, Binfeng Lu, Zongbi Guo, Esther Giehl, Mathilde Feist, Enyong Dai, Weilin Liu, Walter J. Storkus, Yukai He, Zuqiang Liu, David L. Bartlett

**Affiliations:** 1 UPMC Hillman Cancer Center, Pittsburgh, PA USA; 2 Department of Surgery, University of Pittsburgh School of Medicine, Pittsburgh, PA USA; 3 Department of Immunology, University of Pittsburgh School of Medicine, Pittsburgh, PA USA; 4 Fujian Tianjian Pharmaceutical Co. Ltd., Sanming, Fujian China; 5 Department of Dermatology, University of Pittsburgh School of Medicine, Pittsburgh, PA USA; 6 Georgia Cancer Center, Medical College of Georgia, Augusta University, Augusta, GA USA

## Abstract

Cancer vaccines and oncolytic immunotherapy are promising treatment strategies with potential to provide greater clinical benefit to patients with advanced-stage cancer. In particular, recombinant vaccinia viruses (VV) hold great promise as interventional agents. In this article, we first summarize the current understanding of virus biology and viral genes involved in host-virus interactions to further improve the utility of these agents in therapeutic applications. We then discuss recent findings from basic and clinical studies using VV as cancer vaccines and oncolytic immunotherapies. Despite encouraging results gleaned from translational studies in animal models, clinical trials implementing VV vectors alone as cancer vaccines have yielded largely disappointing results. However, the combination of VV vaccines with alternate forms of standard therapies has resulted in superior clinical efficacy. For instance, combination regimens using TG4010 (MVA-MUC1-IL2) with first-line chemotherapy in advanced-stage non-small cell lung cancer or combining PANVAC with docetaxel in the setting of metastatic breast cancer have clearly provided enhanced clinical benefits to patients. Another novel cancer vaccine approach is to stimulate anti-tumor immunity via STING activation in Batf3-dependent dendritic cells (DC) through the use of replication-attenuated VV vectors. Oncolytic VVs have now been engineered for improved safety and superior therapeutic efficacy by arming them with immune-stimulatory genes or pro-apoptotic molecules to facilitate tumor immunogenic cell death, leading to enhanced DC-mediated cross-priming of T cells recognizing tumor antigens, including neoantigens. Encouraging translational and early phase clinical results with Pexa-Vec have matured into an ongoing global phase III trial for patients with hepatocellular carcinoma. Combinatorial approaches, most notably those using immune checkpoint blockade, have produced exciting pre-clinical results and warrant the development of innovative clinical studies. Finally, we discuss major hurdles that remain in the field and offer some perspectives regarding the development of next generation VV vectors for use as cancer therapeutics.

## Background

Humankind has accumulated a rich and extensive clinical experience with vaccinia virus (VV) due to its successful use as a smallpox vaccine. Since the late 1980s, investigators have been harnessing recombinant DNA technology, to explore the utility of recombinant VV and other poxviruses as expression vectors for the purpose of active immunization in the setting of cancer and infectious disease [1]. VV vectors have been extensively studied in pre-clinical tumor models and in many clinical trials for treatment of patients with advanced-stage solid cancers. Despite low rates of objective clinical responses, investigators have learned many important lessons, allowing for the evolution of improved strategies for application in the future [1]. VV has also been systematically explored as an oncolytic virus (OV) over the past 20 years. Among the three oncolytic VVs tested in cancer patients, Pexa-Vec showcases the clinical development of such an OV and is currently being evaluated in a global phase III clinical trial for patients with hepatocellular carcinoma (HCC).

These are indeed exciting times for cancer immunotherapy, as the field is rapidly progressing, fueled by consistent evidence of therapeutic efficacy and durable clinical benefit amongst a subset of treated patients [2–4]. Cancer vaccines and oncolytic immunotherapy represent some of the most promising immunotherapy regimens. Many classic cancer vaccines have utilized non-replicating viruses as vectors to express tumor antigens and/or immune-modulatory molecules [1]. OVs function to kill cancer cells and associated stromal cells through multiple mechanisms, leading to DC-mediated activation of protective anti-tumor immunity. In 1999, Toda et al. demonstrated that an oncolytic herpes simplex virus was capable of inducing specific anti-tumor immunity via a process that they termed as an “in situ cancer vaccine” [5]. We and others have reviewed the concept of using OV as a unique type of cancer vaccine [6, 7] and the likely superior benefits that would be associated with integrating OVs into combination immunotherapies for improving objective clinical response rates [8, 9].

## Biology of vaccinia virus

Poxviruses are comprised of two subfamilies containing at least 46 species: *Chordopoxvirinae* (those infecting vertebrates) and *Entomopoxvirinae* (those infecting insects) [10]. VV, the species of interest in this review, is a member of the orthopoxvirus genus of the *Chordopoxvirinae* subfamily. As smallpox vaccination became widespread throughout the world over the past 200 years, research- and clinical-centers have produced and maintained viruses in different ways, resulting in differential viral characteristics, pathogenicity and host ranges (i.e. different strains of virus). VV has a linear, double-stranded DNA genome approximately 190 kb in length, which encodes about 200 genes. Physically, the virus particle is the shape of a brick, averaging 270 × 350 nm in size.

**Fig. 1:**
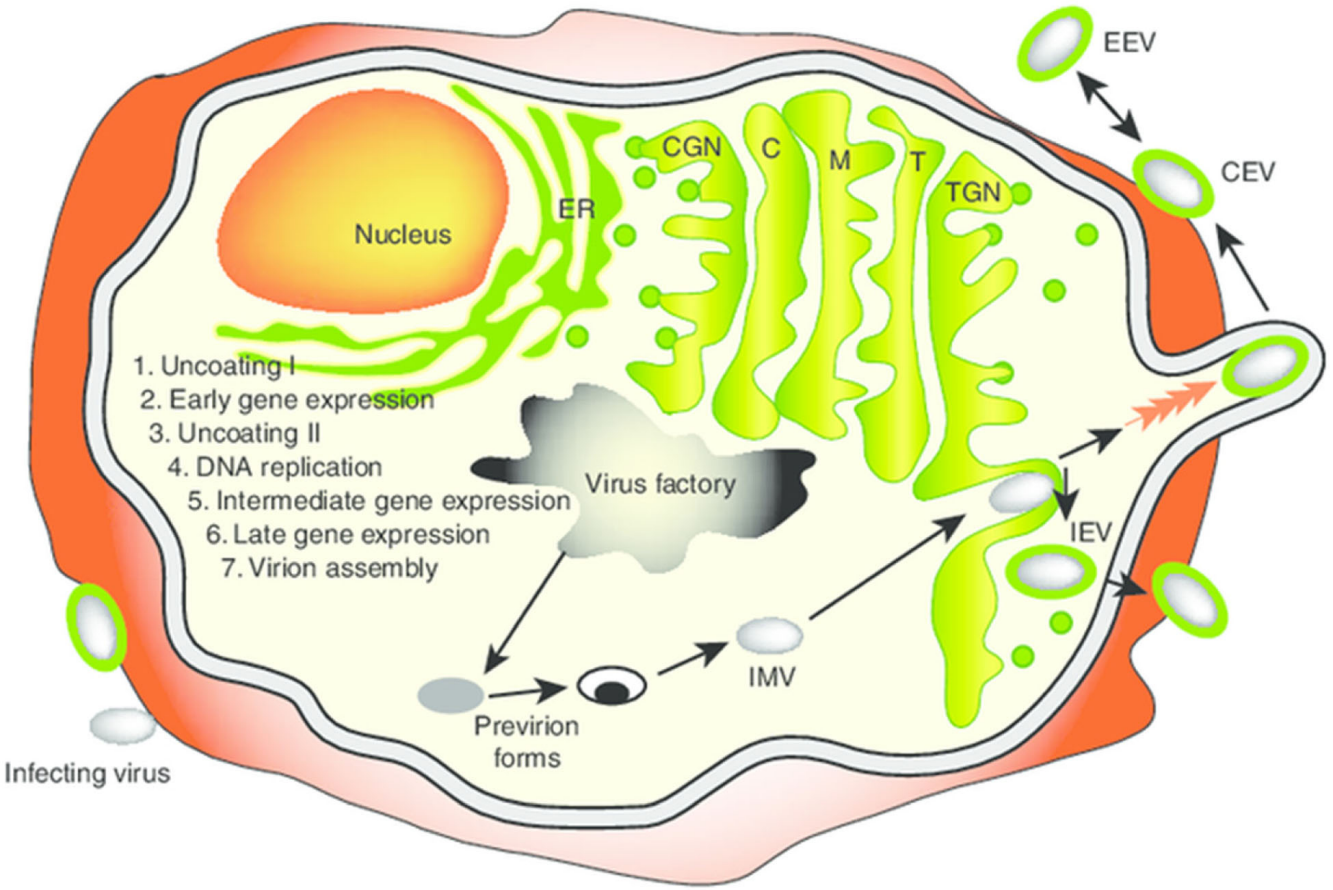
Vaccinia virus life cycle. A diagram of the infected cell with an exaggerated view of the cellular compartments, including the ER (endoplasmic reticulum), CGN (cis-Golgi network), C (cis-Golgi), M (medial-Golgi), T (trans-Golgi) and TGN (trans-Golgi network), is shown. Also shown are the major stages of the viral life cycle. Following late gene expression, pro-virion forms assemble to form the IMV. The IMV targets the TGN and, following envelopment, the IEV is formed. IEVs are propelled to the cell surface by polymerization of actin filaments. Once released, the virus may remain attached to the membrane as a CEV or be released into the medium as an EEV. CEV: cell-associated enveloped virus; EEV: extracellular enveloped virus; IEV: intracellular enveloped virus; IMV: intracellular mature virus. This figure was adapted from Grosenbach DW, Hruby DE. *Front. Biosci*. (1998) 3:d354–364 [174] with permission

The entire VV life cycle occurs within the cytoplasm of mammalian cells (Fig. 1). Cell entry occurs by virion fusion with the host cell membrane [11]. VV contains an outer envelope as well as an internal membrane and incorporates enzymes required for initiation of viral transcription post-infection. Viral transcription can be classified into three stages - early, intermediate, and late – with each increment involving its own specific promoters and transcription factors [12]. In the early phase, enzymes and other components needed for the process of early transcription are contained within the viral core along with viral genomic DNA [12]. A DNA-dependent RNA polymerase is also contained within the viral core, leading to the synthesis of early messenger RNA. Translation of this RNA yields early stage proteins involved in the uncoating of viral DNA, DNA replication, and transactivation of intermediate mRNA. While initial RNA transcripts can be detected within 20 min, the entire replication cycle is complete within approximately 1 h. VV replication and progeny assembly take place exclusively in the cytoplasm of infected cells at discrete foci in endoplasmic reticulum (ER)-enclosed cytoplasmic mini-nuclei [13], known as “poxvirus factories”. VV replication utilizes the origin of DNA replication located near the end of the genome through the leading/lagging strand replication mode [14]. In this regard, VV activates cytoplasmic ATR early during infection and before genome uncoating, which promotes VV genome replication [15]. Intermediate mRNA is then expressed and encodes for late transactivators, leading to late mRNA synthesis. Late proteins include structural proteins for membrane formation and early transcription factors, which are incorporated into new progeny virus particles. The hijacking of the host translation apparatus inside the virus factories by the virus contributes to enhanced viral replication and to the suppression of host protein synthesis, thereby facilitating viral subjugation of infected cells [16]. In fact, virus induces a profound cytopathic effect soon after viral entry, as the early viral enzymes completely shut down host cell functions. Between 4 and 6 h after infection, host protein synthesis is almost completely inhibited, facilitating the efficient expression of viral genes and viral replication. Remarkably, approximately 10,000 copies of the viral genome are made within 12 h post-infection [17].

Several key viral genes, proteins, and molecular and cellular mechanisms are involved in productive viral infection, replication, virion assembly, and the spreading of progeny viruses. Viral entry into cells is mediated by an entry-fusion protein complex consisting of eight viral proteins: A16, A21, A28, G3, G9, H2, J5 and L5 [14]. All eight proteins that make up the entry-fusion complex are conserved in all poxviruses, suggesting non-redundant functions and an evolutionarily-conserved entry mechanism. VV possesses two infectious forms: the intracellular mature virus (IMV) and the extracellular enveloped virus (EEV), also referred to as the MV and EV forms, respectively. It is intriguing to note that attachment to the cell surface differs between IMV and EEV. The IMV form contains several other proteins in its outer envelope, including A17L, A27L, and D8L [18], which likely modulate viral attachment. A27L mediates VV interaction with cell surface heparan sulfate [19, 20], with viral infection inhibited by up to 60% in the presence of soluble heparin [19]. The EEV form is responsible for cell-to-cell spread and long-range transmission of VV in vivo [21]. EEV-specific proteins are encoded by the genes A33R, A34R, A36R, A56R, B5R, and F13 L [10, 18].

The first stage in the formation of infectious particles is the development of viral crescents composed of lipid and viral proteins, with recent evidence suggesting the ER as the origin [22]. These crescents then coalesce into immature virus that lack infectivity. Immature virus becomes IMV by condensation of the core and processing of core proteins. IMV is transported to sites where it becomes wrapped with two additional membrane layers derived from components scavenged from the *trans*-Golgi network. These membranes are modified by the inclusion of virus-encoded proteins. These wrapped, intracellular, enveloped viruses traffic along microtubules to the cell surface, where the outer membrane fuses with the plasma membrane, thus exposing the viruses on the cell surface. IMV particles are transported to the cell periphery on microtubules, where they fuse with the plasma membrane to form cell-associated enveloped virus (CEV). Two intracellular enveloped virus (IEV)-specific proteins, F12 L and A36R, along with cellular microtubules (as motors), mediate IEV transport to the cell periphery [23].

Upon reaching the plasma membrane, VV switches from microtubule-dependent transport to the formation of actin tails required for cell-to-cell spread of virus [24]. This process is dependent on phosphorylation of the EEV protein A36R, a process mediated by multiple families of tyrosine kinases [24–26]. The EEV form may play a role in the rapid spread of VV and its wide host range. The A34R protein helps to maintain the virus particle on the cell surface. The WR strain exists almost exclusively as a cell-associated virus [27], while the IHD-J strain produces quantitatively more of the EEV form of the virus [28]. Further studies show that the A34R gene from the IHD-J strain is responsible for increased EEV production, with a codon mutation (K151E) sufficient to transfer a comet-forming phenotype to the WR virus [29]. Our recent data suggest that a single mutation, K151E, in the A34R protein results in an increase in both EEV release and total progeny virus production from infected cancer cells [30].

VV has evolved three intriguing mechanisms to promote viral spread. The first one is for cell-to-cell spread. Viral protein F11 promotes cell-to-cell spread by acting as a scaffold, using its PDZ domain to unite myosin IXa and RHOA, which inhibits RHOA signaling and ultimately promotes viral spread [31]. The other two mechanisms are for distant spread. One is the production and release of the EEV form [32], while the other is the repulsion of superinfecting virions [33]. Smith et al. recently showed that VV spreads across one cell every 75 min, four-fold faster than its replication cycle permits. The authors found that newly infected cells express two surface viral proteins (A33 and A36) that mark cells as infected and, via exploitation of cellular machinery, induce the repulsion of superinfection virions towards uninfected cells. During this process, protein B5 on the membrane of the EEV particle is required for repulsion of superinfection virions [34]. Improved strategies taking advantage of these unique mechanisms may further improve therapeutic viral spread.

In summary, genetic manipulations of the viral genome may lead us to advances in the development of next generation VV vectors for implementation in cancer therapies.

### Further improvements of viral vectors for use as cancer vaccines and/or OVs

There are numerous advantages and disadvantages to the use of VV in cancer vaccine formulations or as oncolytic agents. Table 1 compares some of the properties of VV, herpes simplex virus (HSV), and coxsackievirus, revealing similarities and differences that impact their clinical utility. All three viruses induce immunogenic tumor cell death, which is beneficial to the development of adaptive therapeutic immunity. In terms of generating mutations in the host genome, HSV carries more risk, as it replicates in the host cell nucleus, and carries the possibility for integration into the host genome [35]. Coxsackievirus is a positive-sense single-stranded RNA virus with two weaknesses that contraindicate its use as a vector. First, it is relatively difficult to clone a gene into this viral genome. Second, the size of foreign insert DNA is very limited, with an upward limit of 300-bases permissible for stable recombinant. Indeed, the use of VV or HSV as vectors (instead of CVB3) is warranted in order to express large single transgenes or the coordinate expression of multiple transgenes.

**Table 1:** A comparison of three oncolytic viruses: strength and pitfalls

Virus	VV	Herpes simplex virus (HSV)	Coxsackievirus A21, B3 (CVA21 and CVB3)
Genome	dsDNA (~ 180-Kb, 200 genes)	dsDNA (~ 152-kb, 80 genes)	(+) ssRNA (~ 7.4-Kb, one polyprotein)
Capacity of inserted DNA	25–40 Kb	30–40 Kb	300 bases for stable recombinant
Tumor selectivity (once inside the cells)	Pexa-Vec: selectively replicates in and destroys cancer cells driven by genetic pathways commonly activated in cancers.	T-VEC: two mutations make up cancer selectivity with activated Ras and high endogenous ribonucleotide reductase	Aberrant signaling pathways within tumor cells On cell surface: CVA21: ICAM-1 dependent. CVB3: CAR dependent
Life cycle	Cytoplasm (no risk of integration)	Nucleus (more risk of integration)	Cytoplasm
Mechanisms of cell death	Apoptosis and necroptosis (ICD)^a^	Apoptosis, necrosis, and pyroptosis (ICD)	Immunogenic apoptosis, autophagy (ICD)
Immunogenicity	High	High	High
Transgene expression	High	High	High
Clinical trial stage	Phase III for liver cancer	T-VEC approved for melanoma	Phase II study in advanced melanoma (CVA21)

^a^*ICD* immunogenic cell death

The advantages of VV may include but are not limited to: (1). Its efficient life cycle, producing mature progeny virus in just 6 h; (2). Its three mechanisms of viral spread, which ensure fast, efficient dissemination of the virus; (3). Its large viral genome, enabling its acceptance of large foreign DNA inserts of up to 40-kb; (4). Its lack of promoting disease in healthy humans; and (5). Extensive clinical experience and knowledge of the virus due to its use as a smallpox vaccine. On the flip side, the viral genome encodes about 200 viral genes, 50% of which have unknown functions, which provides a level of unpredictability for this virus.

Four major advances have recently been made to recombinant VVs and other poxviruses to improve their utility as cancer vaccines or OVs: 1) Further modification of the viral vectors to make them more immunogenic and thus more potent in eliciting antitumor immunity; 2) Arming the virus with genes encoding specific tumor-associated antigens (TAAs) or neo-antigens for improved induction of specific T cell-mediated immunity, 3) Arming them with immunostimulatory molecules to further enhance their immunogenicity, and finally, 4) Combining these vectors with systemic immunomodulatory regimens.

1. Further modification of VV viral vectors for improved immunogenicity.

The key viral genes to be manipulated are those whose products interact with the host leading to altered immunogenicity [36]. In Table 2, we list about 20 virus-encoded genes whose products modulate innate and adaptive immunity. These represent cogent targets for genetic manipulation to enhance viral vector immunogenicity. Since a comprehensive discussion for all of these molecules is not possible in this forum, we have chosen to discuss a limited number of “high-priority” targets.

**Table 2:** Vaccinia virus (VV) encodes multiple genes whose products modulate immune responses

Viral genes	Key function	Relevant findings	References
A41L	chemokine binding protein	Deletion of A41L enhances VV immunogenicity and vaccine efficacy	[175]
A44L	3beta-HSD enzyme (v3beta-HSD)	A44L promotes steroid synthesis	[41, 176]
A46R	TLR inhibitor and putative IL-1 antagonist	A46R is an inhibitor of the TLR4 signaling pathway	[41]
A49	Triggers Wnt signaling	A49 targets β-TrCP and thus affects multiple cellular pathways, including the NF-κB and Wnt signaling cascades	[40]
A52R	Putative inhibitor of TLR signaling	A52R targets Toll-like receptor signaling complexes to suppress host defense	[38]
A53R	Soluble TNF receptor	The gene deleted virus retains high immunogenicity but replication is attenuated	[177]
B5R	Inhibits complement	Anti-B5 (EV protein) antibody-directed cell lysis via complement is a powerful mechanism for clearance of infected cells	[148]
B8R	IFN-γ soluble receptor	B8R is a type II IFN binding protein	[36, 178]
B13R (SPI-2)	Inhibits IL-1β converting enzyme	B13R is a nonessential immune-modulating gene that has antiapoptotic and anti-inflammatory properties with sequence homology to serine protease inhibitors (serpins)	[179]
B15R	IL-1β soluble receptor	Deletion led to increased dendritic cell, natural killer cell, and neutrophil migration, as well as chemokine/cytokine expression	[36, 38]
B18R	IFN-α/β soluble receptor	B18R encodes a secreted decoy receptor with a broad antagonizing effect against type I IFNs. It is good for viral replication	[180]
C3L (VCP)	Complement control protein (VCP)	VCP modulates adaptive immune responses during infection	[181, 182]
C6	Binds to STA2 and inhibits type I IFN signaling	C6 is a dual function protein that inhibits the cellular responses to type I IFNs and as an inhibitor of IRF-3 activation	[183]
C7L	Antagonizes IRF1-induced antiviral activities	C7L inhibits antiviral activities induced by Type I interferons	[184, 185]
C12L	Binds and inhibits IL-18	C12L promotes virulence by reducing gamma interferon production and natural killer and T-cell activity	[41, 186]
E3L	Binds dsRNA to block PKR activation	E3 protein prevents the antiviral action of ISG15	[187]
F1L	Inhibits cytochrome C	F1L promotes virulence by inhibiting inflammasome activation	[188]
K1L	Inhibits NF-κB activation	K1L supports viral replication in human cells. Deletion of the gene led to a virus that is less pathogenic due to muted innate immune responses, yet still elicits protective immunity	[39]
K3L	The dsRNA-activated protein kinase (PKR) is inhibited by this pseudosubstrate inhibitor	K3L prevents phosphorylation of e1F2α	[189, 190]
K7R	Promotes histone methylation associated with heterochromatin association	K7R is a virulence gene; it inhibits the NF-κB pathway and thus the migration of neutrophil cells. It affects the acute immune response	[37, 38, 191, 192]
M1L	Associates with apoptosome	The current model is that M1L associates with and allows the formation of the apoptosome, but prevents apoptotic functions of the apoptosome	[193]
N1L	Inhibits NF-κB	N1L is a Bcl-2-like anti-apoptotic protein. It inhibits the NK cell response	[194]

Due to the limitation of the number of references that can be cited for this journal, not all relevant papers can be listed

Products of several viral genes inhibit the NF-κB signaling pathways via a range of mechanisms. These genes include A49, A52R, B15R, K1L, K7R, and possibly others [37–40]. Esteban and colleagues have studied three genes, A52, B15, and K7. These gene products act coordinately to inhibit NF-κB. After infection of a VV incorporating a deletion of these three genes, NF-κB is activated, leading to the production of pro-inflammatory cytokine/chemokines which recruit neutrophils (Nα and Nβ), a sub-type of antigen-presenting cells (APCs), into sites of infection. This subsequently sponsors enhanced T cell responses against both the virus and any vector-encoded antigens [37, 38]. Shisler and colleagues showed that VV with a deletion of the K1L gene is less pathogenic, less immunogenic, and less capable of promoting immune cell infiltration in an intradermal model, yet it was still competent to elicit protective immunity [39]. In another study, the authors deleted the viral genes A44L, A46R, and C12L from the MVA genome. This modified MVA showed enhanced immunogenicity via a mechanism involving innate immune cell activation, leading to corollary generation of specific T-cell responses [41]. VV A49 protein targets the cellular E3 ubiquitin ligase β-TrCP, with the latter responsible for the ubiquitylation and consequent proteasome-mediated degradation of IκBα, resulting in the release of the NF-κB heterodimer. The enzyme ubiquitylates multiple cellular substrates, including the transcriptional activator β-catenin. In a recent study, expression of viral A49 was shown to cause an accumulation of β-catenin and β-TrCP-dependent activation of Wnt signaling [40].

Another class of interesting targets are the viral genes involved in cell death [42, 43]. Previously, we showed that deletion of the Spi-2/Spi-1 genes from VV renders it highly-attenuated in normal tissue, yet it retains replication competence in cancer cells and functions as a potent oncolytic VV [44]. At the same time, Yilma and colleagues showed that the same deletion of genes, with a coordinate enforcement of IFN-γ expression, leads to improved VV induction of immune responses in the absence of detectable replication in normal tissues. As a consequence, such vectors may prove to be extremely safe and effective when applied as vaccines against cancer and other diseases [45]. While not all types of cancer cell death are considered immunostimulatory, the concept of “immunogenic cell death” has been recently advanced [46]. Necroptosis is one of the few types of cell death that classifies as immunogenic cell death. In this regard, it is highly interesting to note that the immune evasion protein E3 encoded by VV inhibits DAI-dependent necroptosis. VV with a deletion of the Zalpha domain of E3 induced rapid RIPK3-dependent cell death in IFN-treated L929 cells [47]. This virus is attenuated in vivo.

2). Induction of systemic antitumor immunity via STING and Batf3-dependent dendritic cells.

Mammalian cells have evolved defense mechanisms against infection, with rapid detection of microbial agents. The STING (stimulator of interferon genes)-controlled innate immune pathway mediates cytosolic DNA-induced signaling events. The knowledge we have gained through this particular signaling pathway may open new ways to induce novel immunization and therapeutic strategies to treat cancer [48].

In this regard, a recent study on inactivated VV raised some intriguing notions relevant to the design of prospective cancer vaccine strategies. The MVA strain of VV is an attenuated poxvirus that has been engineered for use as a cancer vaccine. It triggers type I IFN production in conventional dendritic cells (cDCs) via a cGAS/STING-mediated cytosolic DNA-sensing pathway [49]. When cDCs were infected with heat- or ultraviolet-inactivated MVA, it led to higher levels of interferon production versus MVA alone as a consequence of STING activation [50]. The injection of inactivated MVA directly into tumors led to the generation of therapeutic adaptive antitumor responses in murine melanoma and colon cancer models. In addition, the authors suggest that both cytosolic DNA sensing and Batf3-dependent CD103^+^/CD8α^+^ DCs are essential to the antitumor efficacy of this mode of cancer immunotherapy [50]. Another group also showed that STING agonist-formulated cancer vaccines can cure established tumors refractory to PD-1 blockade [51].

3). Arming VV with genes for enhanced immunogenicity, including tumor antigens and co-stimulatory molecules.

To improve their immunogenicity, investigators have armed viral vectors with genes encoding specific TAAs, neoantigens and/or immunostimulatory molecules. Several groups have generated recombinant VVs expressing various cytokines and used them as cancer vaccines in the 1990s. These early studies strongly support the concept that VVs expressing a tumor antigen and a Th1-stimulatory cytokine function as potent cancer vaccines, leading to the translation of these approaches into the clinic for the treatment of cancer patients.

One of the key issues in cancer vaccines has been how to most effectively enhance the inherently-weak immunogenicity of tumor-associated antigens (TAAs) in the face of intrinsic viral proteins that are highly-immunogenic. When provoking an immune response using viral vectors, weaker epitopes derived from TAAs may be ignored by the adaptive immune response based on a biased focus on xenogeneic viral proteins [52]. This dichotomous response needs to be overcome in order for viral vectors to be optimally exploited in effective cancer vaccines. A first strategy to correct this imbalance in the immune response is to limit immune responses to the viral antigens while boosting the immune response to tumor antigens by benefiting from lessons learned about viral pathogenicity. Many viruses have acquired inhibitors that target essential stages of the MHC class I antigen presentation pathway. For example, ICP47 encoded by HSV strongly downregulates MHC class I antigen-restricted presentation by blocking the ability of transporter associated with antigen processing (TAP) proteins from conveying peptides into the ER for loading into nascent MHC class I complexes, thus limiting CD8^+^ T cell recognition of infected target cells. In one strategy [53], the authors developed VVs expressing either ICP47 alone or together with an ER-targeted Melan-A/MART-1_27–35_ peptide epitope and used them to infect APCs. Infected APCs were defective in their ability to present TAP-dependent MHC class I-restricted viral epitopes to CD8^+^ T-cells even though HLA class I molecules were expressed, as a result of ICP47-dependent suppression of molecules important for aggregate TCR signaling (such as CD80, CD44, and MHC class II). However, a significantly enhanced CTL response can be detected in cultures co-stimulated with rVV-MUS12 expressing an ER-targeted (TAP-independent) tumor epitope, an approach suitable for translation as a cancer immunotherapy [53].

Significant attention has been devoted to the engineering of VV to encode immune costimulatory molecules, as at least two signals are required for the productive activation of naive T cells by antigen-bearing target (stimulator) cells: an antigen-specific signal (signal 1) delivered through the T-cell receptor, and a costimulatory signal delivered through the T-cell surface molecule CD28 (signal 2). In 1995, Schlom and others showed that a mixture of two VVs, one expressing a tumor antigen CEA and the other expressing co-stimulatory molecule B7.1 (rV-CEA and rV-B7), led to not only to the generation of optimal CEA-specific T cell responses, but also to the prevention of CEA^+^ tumor establishment in mice [54]. This same team of investigators then designed poxvirus vectors encoding a TRIad of COstimulatory Molecules (B7–1/ICAM/LFA-3, termed TRICOM). They showed that these vectors induced a more robust activation of T cells when compared to cells infected with homologous virus encoding any one or two of these costimulatory molecules [55]. This study had broad implications in vaccine design/development and led to the performance of several clinical trials that will be discussed in greater detail later in this article.

A different regimen to improve the immunogenicity of TAAs is to apply a heterologous prime-boost regimen in cancer vaccines. By using two “mis-matched” poxviruses, PANVAC™-VF combines MVA and fowlpox viral vectors, expressing the two human antigens CEA and MUC1 and TRICOM costimulatory molecules in both vectors in order to elicit superior tumor antigen-specific immune responses [56].

Cerullo and others have recently developed a personalized cancer vaccine platform implementing clinically-relevant oncolytic enveloped viruses that can drive the expansion of responses against tumor antigens. By physically attaching tumor antigen-derived peptides onto the viral envelope of VV and HSV-1, the authors were able to induce strong T cell-specific immune responses. They demonstrated that OVA SIINFEKL-peptide-coated viruses and gp100-Trp2-peptide-coated viruses, respectively, promoted therapeutic CD8^+^ T cell responses against B16.OVA and B16-F10 melanomas in mice [57].

4). Combination with alternate immunotherapy approaches, including immune checkpoint blockade.

To bolster the immunostimulatory capacity of VV over the past several years, many have chosen to combine virus administration with agonists of immune co-stimulatory molecules or antagonists of immune co-inhibitory molecules (i.e. checkpoint blockade). For instance, vaccination with VV expressing a costimulatory molecule 4-1BBL (rV-4-1BBL) combined with host lymphodepletion (to remove regulatory cells and provide “space” for homeostatic T cell expansion) led to enhanced therapeutic activity versus vaccination with VV alone [58].

TG4010 is a MVA expressing human mucin1 (MUC1) and IL-2. Preclinical combination immunotherapy studies have been performed to further improve its efficacy. Sequential administration of a MVA-MUC1 cancer vaccine and the TLR9 ligand, Litenimod, improved local immune defense against tumors [59]. In another study, first MVA-βGal and MVA-MUC1 treatments were used to treat mice with established CT26 colon carcinomas. Treatment with MVA vectors led to the accumulation of CD3^dim^CD8^dim^ T cells, with two subpopulations characterized as short-lived effector cells and early effector cells (EECs) secreting IFNγ and granzyme B, and translocating CD107a to the cell surface (as a surrogate to lytic granule release) upon antigen-specific peptide stimulation. However, EECs were characterized with high expression levels of the immune checkpoint molecule PD-1. In addition, tumor growth in the diseased lung correlated with PD1^+^ T*reg* cells that was partially reduced after TG4010 treatment. In the late stages of disease, PD-L1 was detected on cancer cells and immune cells, including CD4^+^ T cells (including T*reg* cells), CD3^+^CD8^+^ and CD3^dim^CD8^dim^ T cells, natural killer (NK) cells, myeloid-derived suppressor cells, and alveolar macrophages. When PD-1 blockade using specific antagonist antibodies was applied several days after TG4010 treatment, therapeutic benefits associated with viral therapy were enhanced [60]. In a third study, MVA-BN-HER2 poxvirus-based active immunotherapy administered alone or in combination with CTLA-4 checkpoint blockade was investigated in the treatment of CT26-HER-2 lung metastases in mice. MVA-BN-HER2 immunotherapy significantly improved median overall survival. However, when the virus was combined with immune checkpoint blockade, therapeutic benefits were dramatically improved [61]. These data support ongoing clinical evaluation of TG4010 immunotherapy in combination with nivolumab (anti-PD1) or other combinations of VV-based cancer vaccine plus immune checkpoint blockade.

### Clinical studies of VV as cancer vaccines

The utility of VV and other poxviruses (mostly MVA and fowl poxvirus) as vehicles for cancer vaccines have been actively investigated over the last 30 years. Representative successful clinical studies are listed in Table 3. In the 1980s and 1990s, investigators explored the concept of using wild-type vaccinia-infected melanoma cell lysates for cancer vaccines. In 1995, a phase III, randomized, double-blind multi-institutional trial of wild-type vaccinia-infected melanoma cell lysates-active specific immunotherapy for patients with stage II melanoma showed no difference in disease-free interval or overall survival when compared to naked VV delivery [62]. As a consequence of such findings, interest in such approaches has faded, but the birth of recombinant DNA technology has since revived the use of VV recombinants as cancer vaccines. Mastrangelo et al. conducted the first clinical trial using a recombinant VV expressing GM-CSF in melanoma patients and published their results in 1999 [63]. Later, a phase I/II trial for melanoma patients was conducted using recombinant VV expressing ER-targeted HLA-A0201-restricted melan-A/MART-1_27-35_, gp100_280–288_, tyrosinase_1–9_ epitopes together with CD80 and CD86 proteins [64]. In this case, only weak anti-tumor immune responses were observed in most patients. Interestingly, when a VV vector coordinately expressing multiple antigen epitopes and two co-stimulatory molecules B7.1 and B7.2 in the context of systemic GM-CSF was administered to melanoma patients, specific CTLs against melanoma-associated antigens were rapidly induced in vivo [65]. In 2005, Kaufman, Marincola and others treated melanoma patients with a VV vector expressing B7.1. A standard two-dose-escalation phase I trial was conducted in 12 patients. The approach was well-tolerated, with direct injection of B7.1-expressing VV into melanoma lesions resulting in the development of both local and systemic immunity in association with objective clinical responses. Increased frequencies of gp100- and MART-1-specific CD8^+^ T cells were identified in patient peripheral blood in ELISPOT assays [66]. Similarly, VV expressing the TRICOM (rV-TRICOM) was used to treat 13 patients with metastatic melanoma. Vaccination was well-tolerated, with only low-grade injection site reactions associated with mild fatigue and myalgia observed. Overall, there was a 31% objective clinical response, with one patient achieving a durable complete response for 22 months [67].

**Table 3:** Recombinant vaccinia virus (VV) vectors as cancer vaccines: representative clinical studies

Name	VV strains or other poxvirus	TAA	Immunostimulatory gene or agents	Clinical trial stage and type of cancer	Immunological responses and clinical outcomes	References
TroVax	MVA	5 T4	A variety of agents (such as IL-2, IFN-α, sunitinib)	Phases II and III (*n* = 733) Metastatic renal cancer	(1). Patients with good prognosis receiving vaccine + IL-2 had improved overall survival when compared to IL-2 alone. (2). Association between 5 T4-specific (but not MVA) antibody responses and enhanced survival.	[73, 195]
VV with A0201- restricted epitopes	MVA	Epitopes from gp100, MART-2 & tyrosinase	B7.1 and B7.2 (CD80 and CD86)	Phase I, II Melanoma	Direct injection into lymph node, or given as a prime followed by peptide boosting; both gave antigen-specific CD8^+^ T cell responses. No overall survival benefit.	[64, 196]
TG4010 + chemo	MVA	MUC1	IL-2	Phase 2b Non-small cell lung cancer	TG4010 plus chemotherapy seems to improve progression-free survival relative to placebo plus chemotherapy. Because the primary endpoint was met, the trial will continue into phase III.	[82]
MVA-brachyury-TRICOM	MVA	Brachyury	TRICOM [B7.1, ICAM-1, LFA3]	Phase I (*n* = 38) Advanced cancer patients	Brachyury-specific T-cell responses were observed at all dose levels and in most patients.	[197]
PROSTVAC	VV prime and fowlpox boost	PSA	TRICOM [B7.1, ICAM-1, LFA3]	Phase II Prostate cancer	Increased PSA-specific CTL responses, particularly with GM-CSF or IL-2. In prostate cancer, an increase in progression-free survival was observed.	[78, 79]
PANVAC + chemo (docetaxel)	PANVAC (VV and fowlpox)	CEA and MUC1	Just PANVAC or none (chemo alone)	Phase II (*n* = 48) patients with metastatic breast cancer	Combination of PANVAC with docetaxel provides a clinical benefit. The median progression-free survival was 7.9 months in the combination group vs. 3.9 months in the chemo group.	[86]

More advanced clinical studies have been performed over the past decade. Four sets of phase I-III clinical studies (with phase III trials performed in two cases) have been completed in cancer patients, advancing the clinical utility of MVA-5 T4, rV-PSA, TG4010 and PANVAC.

In the first set of trials, an MVA encoding the tumor antigen 5 T4 (MVA-5 T4, termed TroVax) was used as a vaccine against cancers expressing this antigen. The human oncofetal antigen 5 T4 (h5 T4) is a transmembrane glycoprotein overexpressed by a wide spectrum of cancers, including colorectal, ovarian and gastric carcinomas, but with only limited expression in normal tissues. Preclinical studies supported the effectiveness of MVA-5 T4 in a range of tumor models [68]. The first two trials were conducted in colorectal cancer patients, either used alone or in conjunction with chemotherapy. The first trial showed that vaccination with TroVax was safe. Specific immune responses against 5 T4 were induced in treated patients, and anti-5 T4 antibody responses were found to correlate with evidence of disease control [69]. When co-administered with chemotherapy, TroVax induced robust immune responses. Not too surprisingly, 5 T4 (tumor)-specific immune responses, but not MVA (viral)-specific immune responses, were found to correlate with clinical benefit [70].

In patients with metastatic renal cell carcinoma, vaccination with TroVax did not improve objective response rates vs high-dose IL-2 monotherapy, but vaccination resulted in disease stabilization in association with an increased ratio of 5 T4-specific T effector cell-to-T*reg* cells [71]. In a similar phase II trial with TroVax alone or administered in combination with IFN-alpha, treatments were well-tolerated in all patients. Despite high frequencies of 5 T4-specific T cells being developed in patients post-treatment, no objective clinical benefit was observed in this study [72]. In 2010, a randomized, double-blind, placebo-controlled, phase III study for vaccination of metastatic renal cancer patients with MVA-5 T4 was completed. In this study, 733 cancer patients (365 MVA-5 T4 and 368 placebo) were recruited. Between the two arms, no significant difference in the incidence of adverse events, serious adverse events or overall survival was observed. In a subset of patients, however, the magnitude of 5 T4-specific antibody responses induced by the vaccine was found to be associated with extended patient survival [73].

A series of clinical studies in advanced-stage prostate cancer patients have been performed using MVA encoding human prostate-specific antigen (rV-PSA). The first phase I trial was completed in 2000, with rV-PSA found to be safe and capable of eliciting specific T-cell responses against PSA and extended time-to-progression in a minority of treated patients [74]. In 2002, this vaccine was applied in a phase I trial in patients with metastatic androgen-independent prostate cancer, with some patients developing expansions in their PSA-specific T cell populations in peripheral blood after vaccination [75]. In 2006, Kaufman led a Cooperative Oncology Group sponsored phase II study using a prime/boost vaccine strategy implementing VV and fowlpox virus expressing human PSA. This regimen was well-tolerated, with a significant percentage of patients remaining free of detectable (serum) PSA and clinical progression after 19 months of follow-up. Nearly half of treated patients demonstrated evidence of vaccine-induced anti-PSA T cell responses [76]. Based on this substantial foundation, a new study was designed to improve the vaccines by using both VV and fowlpox vectors coordinately expressing TRICOM and PSA antigen. This phase I study demonstrated that vaccination with PROSTVAC-V and PROSTVAC-F combined with TRICOM is both well-tolerated and competent to promote specific immune response after vaccination with VV [77]. Furthermore, in a phase III trial using the same regimen of two vectors expressing four genes (TRICOM and PSA), the authors examined patient overall survival and immunological/prognostic factors associated with overall survival benefit in the setting of metastatic castrate-resistant prostate cancer. PROSTVAC-VF-based immunotherapy was fond safe and effective in reducing the patient death rate by 44%, leading to an 8.5-month improvement in median overall survival [78]. Finally, the authors concluded that patients developing strong PSA-specific T-cell responses were more likely to live longer [79].

The vaccine TG4010, an MVA vector expressing MUC1 and IL-2, has been evaluated in 2 randomized clinical trials in combination with first-line chemotherapy for treatment of patients with advanced-stage non-small-cell lung cancer [80, 81]. The combination was found safe and effective in improving progression-free survival at 6 months and the proportion of patients achieving clinical responses. In a recent clinical study, Quoix et al. reported their results for the Phase 2b portion of a randomized, double-blind, placebo-controlled phase 2/b/3 trial, supporting the conclusion that TG4010 plus chemotherapy likely improves the progression-free survival of patients when compared to treatment with a placebo plus chemotherapy. This trial is being continued into a phase III study, as the primary endpoint had been met [82].

The fourth regimen (PANVAC™-VF) combines MVA and fowlpox viral vectors, with each virus co-expressing the two antigens CEA and MUC1, in addition to TRICOM costimulatory molecules [56]. This prime-boost regimen has now been evaluated in three clinical trials for patients with advanced-stage carcinomas, including those of the breast, ovary and pancreas. Although TAA-specific immune responses were generated in some patients, minimal objective clinical responses were observed [83–85]. More encouraging data were obtained in a clinical trial of combined treatment with PANVAC and docetaxel in metastatic breast cancer patients, where clinical benefits were noted [86].

What have we learned from these vaccine clinical trials using VV and fowl poxvirus-based vectors expressing cancer-associated antigens? First, all the vaccine-targeted antigens (CEA, PSA, or 5 T4) represent non-mutated sequences, which have typically exhibited low immunogenicity, linked to weak immune responses or to immune tolerance. It’s perhaps then not surprising that we’ve typically witnessed weak anti-tumor immune responses at best amongst treated patients using these modalities. Recent studies have strongly suggested that mutated tumor neo-antigens are appreciably more immunogenic and that immune recognition of neo-antigens is a major factor in the bio-efficacy of immunotherapies in the clinical arena [87]. A recent study identified neoantigens with unique potential to serve as targets for T cell recognition in patients with pancreatic ductal adenocarcinoma. Notably, the authors considered neoantigen “quality” as a biomarker for immunogenic tumors that may guide the application of targeted immunotherapies [88]. Indeed, the recent success of personalized cancer vaccines appears to depend on immune targeting of verified neo-antigens from individual cancer patients [89, 90]. One most recent breakthrough study shows that noncoding regions are the main source of targetable tumor-specific antigens [91]. As a consequence, it would be expected that future trials will incorporate neo-antigen-expressing poxvirus vectors. Second, the heterologous prime-boost regimen remains a well-justified vaccine approach, despite findings that treatment with PANVAC-VF failed to yield objective clinical benefit, potentially suggesting larger rate-limiting issues for other aspects of the treatment (such as the targeting of only weak, self-antigens). Third, in many vaccine studies in the settings of infectious disease and cancer, investigators have selected multi-genic viral vectors for implementations based on suppositions of higher potency and improved efficacy [92–94]. In this context, it would be logical to construct therapeutic VV vectors expressing fusion gene products containing multiple T cell epitopes derived from multiple tumor-associated antigens to expand a broad anti-tumor T cell repertoire capable of providing improved treatment benefit. Fourth, as we now know, the immunosuppressive tumor microenvironment (TME) plays a major role in legislating the outcome of immunotherapy [95]. If a patient presents with an immunologically ‘cold’ tumor, then circulating (vaccine-induced) T cells and other pro-inflammatory immune cells would be challenged to traffic into tumor sites; whereas, if the patient presents with a ‘hot’ tumor, immune checkpoint molecules may prevent the sustained functionality of anti-tumor T cells [96]. As we will discuss later in this article, OVs may be particularly well suited to inflame the TME and to convert immunologically ‘cold’ tumors into ‘hot’ [97, 98]. In his light, we may consider using replicating VVs as cancer vaccines. Finally, the highly immunosuppressive TME expresses multiple immune co-inhibitory molecules, such as PD-1/PD-L1, CTLA-4/CD80, and BTLA/HVEM [99]. Therefore, in the future, a successful cancer vaccine regimen would be expected to include one or more checkpoint antagonists to sustain or expand the pro-inflammatory TME [100].

### VV for oncolytic immunotherapy

We and Fodor’s group were the first to explore the use of genetically-engineered VV as an OV, with research papers published in 1999 and 2000 [101, 102]. During the last 20 years, investigators have created a variety of oncolytic VVs for preclinical studies [103] (Table 4).

**Table 4:** Selective examples of oncolytic vaccinia virus (VV) used in preclinical studies

Virus name	Strain	Transgene	Mode of cell death	Antitumor activities, especially immunity	Tumor models	References
Pexa-Vec (JX-594)	Wyeth (*tk*-)	GM-CSF	Apoptosis and necrosis (ICD)	Tumor cell infection and lysis; antitumor immune response; tumor vascular disruption	hepatocellular carcinoma (HCC) and other cancers	[63, 104, 125]
vvDD-GFP	WR (*tk*−/*vgf*-)	EGFP; (later CD; GM-CSF)	Necrosis and apoptosis; (ICD))	CD11b + cells and CD11b + Ly6G+ cells (dendritic cells and neutrophils)	Breast, colon, and ovarian cancer models	[105, 120]
GLV-1 h68	Lister (deletion of *tk*, *F14.5 L*, *A65R*)	Renilla luciferase-GFP fusion protein, β-galactosidase, β-glucuronidase	Apoptosis and others	Immune defense activation via IFN-stimulated genes (STAT-1 and IRF-7), cytokines, chemokines, and innate immune effector function	Breast cancer and other cancer types	[107, 198]
VG9-GMCSF	Tiantan Guang9 strain (*tk*-)	GM-CSF	Unknown	Antitumor activity and induced tumor-specific immune response	Melanoma	[127]
∆*F4L*∆*J2R*	WR (*F4L*- and *tk*-)	Luciferase	Unknown	Durable tumor-antigen specific cytotoxic T-cell response	Bladder cancer	[110]
CVV	Wyeth strain *tk*- and repeated selection	GFP	Unknown	Complete regression of liver tumorigenicity and metastasis to the colon.	HCC	[115]
deVV5	Chimeric VV from WY, MVA, WR, and COP	*tk* deletion and *fcu1* addition results in deVV5-fcu1	Unknown	Higher tumor selectivity and more viral replication in cancer cells	Not tested yet	[113]
CF33 and CF189	Chimeric parapoxvirus		Unknown	Effective at low viral dose; abscopal antitumor effect	Triple negative breast cancer and colorectal cancer	[114, 123]

#### Further genetic engineering of OV

Manipulations were designed to achieve a higher degree of tumor-selectivity, better efficiency of vector delivery to the tumor, enhanced therapeutic efficacy, and minimized toxicity. The first genetically engineered version of oncolytic VV involved the deletion of the thymidine kinase (*tk)* gene alone [101, 102]. Parato et al. demonstrated that the *tk-*deleted JX-594 (Pexa-Vec) virus selectively replicates in and destroys cancer cells [104]. We have shown that vvDD, an oncolytic VV with a dual deletion of viral genes encoding *tk* and vaccinia growth factor (*vgf*), is highly tumor-selective [105]. Based on the hypothesis that cancer cells often overexpress multiple anti-apoptotic proteins, and are thus more resistant to apoptosis, we deleted two anti-apoptotic viral genes, SPI-1 and SPI-2, resulting in a highly tumor-selective virus (vSP) that retains oncolytic potency [44]. Furthermore, pathway-reinforcing oncolytic VV have been generated that coordinately promote the IFN-beta pathway while deleting the B18R gene product, known to neutralize secreted type-I IFNs [106]. Zhang et al. have also generated an oncolytic VV from the Lister strain with triple insertional mutations in the F14.5 L, J2R (for *tk*), and A56R (encoding hemagglutinin) genetic loci of the viral genome [107]. VV produces a special form of virus particles called EEV, which can evade the neutralizing antibodies and complement-mediated disruption of the virus [36, 108]. The EEV-enhanced strains of VVs displayed enhanced spread within tumors after systemic delivery, resulting in significantly improved antitumor effects, in addition to reduced clearance by neutralizing antibodies commonly developed in experimental models [30, 109].

Recently, new oncolytic VVs have been designed and studied in tumor models. Evans and colleagues have explored another viral gene targeting the nucleotide biosynthesis pathway and developed a new oncolytic VV in which the F4L viral gene encoding ribonucleotide reductase is deleted. This virus is a selective OV and is capable of promoting therapeutic anti-tumor immunity in association with a superior safety index in cancer models [110]. It is interesting to note that both F4L and J2R (*tk*) encode enzymes for nucleotide synthesis. In another study, Esteban and others showed that the WR-Delta4 virus, with a combined deletion of four viral genes that act on metabolic, proliferation, and signaling pathways (A48R, B18R, C11R, and J2R), still effectively mediates anti-tumor benefit while retaining tumor selectivity in vivo. When applied in B16F10 melanoma models, strong viral attenuation, reduced virus dissemination, and efficient inhibition of tumor growth were observed, with a concurrent enhancement in neutrophil infiltration and the induction of tumor antigen-specific immunity [111].

Another strategy is quite unique. VV encodes two de-capping enzymes (D9 and D10) that remove protective caps from mRNA 5′-termini, thus accelerating mRNA decay and limiting activation of host defenses. Mohr and associates showed that D9- or D10-deficient VACV are markedly attenuated in mice, but these VVs are effective when used as oncolytic viruses [112].

Another interesting strategy is to generate novel chimeric poxviruses via homologous recombination of different strains of VV or poxviruses or by natural selection in cancer cells themselves. Erbs and associates mixed four strains of VVs and generated deVV5, a novel chimeric poxvirus with improved oncolytic potency in human cancer cells [113]. Chen and colleagues generated another virus, CF189, by mixing different species of poxviruses; yielding a chimeric OV that is therapeutically effective in triple negative breast cancer models, even at low viral doses [114]. In other studies, Yoo et al. selected an OV designated CVV by repeated selective replication in cancerous tissues, with subsequent deletion of the viral *tk* gene, yielding a therapeutic vector under investigation against metastatic liver cancer [115].

#### Oncolytic VV induces immunogenic cell death

A few years ago, we directly contributed to the conceptual evolution of immunogenic cell death (ICD). ICD was originally proposed by Zitvogel, Kroemer, and others, as a version of immunogenic apoptosis [116]. In 2013, we independently expanded the novel concept from the originally proposed immunogenic apoptosis to include other types of cell death, such as autophagic cell death, necroptosis, and pyroptosis [6, 117]. This expanded concept of ICD has since been widely accepted by the scientific community [46, 118].

**Fig. 2:**
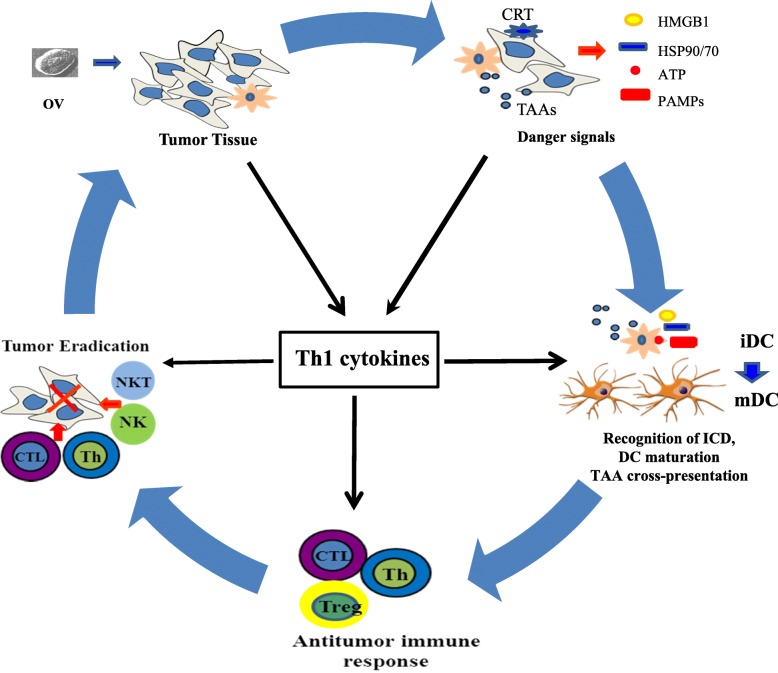
A model of how immunogenic cell death (ICD) and expression of proinflammatory Th1 cytokines from an oncolytic virus (OV) lead to potent antitumor immunity. An OV selectively replicates in tumor or/and stromal cells. This leads to induction of ICD, presenting both “find me” (extracellular HMGB1 and ATP) and “eat me” signals on the cell surface (such as ecto-CRT) to phagocytes. The presented/released danger signals (DAMPs and PAMPs) activate immature DC (iDC) to become mature DC (mDC). Apoptotic bodies and cellular fragments released via ICD are engulfed by APCs, and TAAs are processed into peptides that are presented in MHC class I/II complexes in concert with costimulatory molecules to naive CD8^+^ and CD4^+^ T cells, respectively. Such activated T cells may then expand and undergo polarized differentiation predictable on additional immune-stimulatory molecules expressed by recombinant OV. This figure has been modified from our previous model [6]. HMGB1: high mobility group box 1; DAMP: damage-associated molecular pattern; PAMP: pathogen-associated molecular pattern; APC: antigen-presenting cell; TAA: tumor-associated antigen

Accumulating evidence suggests that oncolytic VVs induce ICD in infected cancer cells and associated stromal cells. VV-induced lytic cell death elicits the release of immunogenic heat shock protein GRP94/gp96 [119]. We showed that the wild-type WR strain and genetically-engineered vSP induced both necrosis and apoptosis, resulting in the release of high mobility group box 1 (HMGB1) from infected and dying cancer cells [44]. Another study showed that vvDD induced necrosis and infected cell release of ATP and HMGB1, two key danger molecules for initiation of the anti-tumor immune responses [120]. Another WR strain-derived OV also exhibited the ability to induce ICD in infected cancer cells [121]. Lister strain *tk*-deleted VV and a chimeric orthopoxvirus induced necroptosis (ICD) in ovarian and colorectal cancer cells [122, 123]. Interestingly, DCs infected by VV also die via ICD, which enhances CD8^+^ T cell proliferation [124]. In summary, oncolytic VVs induce ICD, providing the antigenic fuel for enhanced cross-priming of therapeutic CD8^+^ T cell responses. There remains significant room for improvement in such approaches based on the integration of combination strategies (Fig. 2).

#### VV armed with immuno-stimulatory genes for enhanced antitumor immunity

Investigators have engineered oncolytic VVs with a variety of immunostimulatory genes. For example, VV strains of Wyeth, WR, and Tiantan have been armed with GM-CSF [63, 125–127], a cytokine known to sustain and support the function of DC. As we mentioned earlier, many of the previously created VVs expressing various cytokines used for cancer vaccines in the 1990s turned out to be tumor-selective OVs. Recently, the Wang group has generated a novel vector expressing IL-10 and have demonstrated it to function as an effective therapeutic agent in a murine pancreatic cancer model [128]. Oncolytic VV expressing IL-24 suppresses the growth of lung cancer [129]. We have shown that oncolytic VVs expressing CXCL11 or superagonist IL-15 are also potent inducers of antitumor immunity [130, 131]. Recently, we have modified a VV expressing an membrane-anchored IL-2 to modulate the TME and have effectively treated a variety of murine tumors without evidence of adverse events [132].

Using an alternate novel strategy, Song and colleagues armed an oncolytic VV with a gene encoding a secretory bispecific T-cell engager consisting of two single-chain variable fragments specific for CD3 and the tumor cell surface antigen EphA2 (EphA2-T-cell engager-armed VV (EphA2-TEA-VV)). Administration of EphA2-TEA-VV and adoptive T cell transfer mediated superior anti-tumor benefit when compared to control VV plus T cells in a lung cancer xenograft model. This could represent a promising approach to improve oncolytic immunotherapy [133].

#### Targeting the immunological TME

Advanced cancers display a highly immunosuppressive TME [134]. In order for therapeutic regimens (especially immunotherapies) to work effectively, the TME requires re-conditioning to yield an immunologically favorable theater of operation [135]. The main goals for conditioning regimens are, i.) to enhance the immunogenicity of the tumor tissue in support of improved immune cell recognition and the cross-priming of a diversified therapeutic T cell repertoire and ii.) to recruit and maintain poly-functional anti-tumor innate and adaptive immune effector cells. It is worth pointing out that the virus itself is highly immunogenic and capable of promoting local inflammation. Indeed, recent studies support an operational paradigm in which effective oncolytic therapy and other forms of immunotherapy convert “cold” tumors into “hot” tumors [98, 136, 137].

Such inflammatory conditioning however can lead to compensatory upregulation of regulatory pathways. For instance, local production of pro-inflammatory cytokines can stimulate upregulation in PGE2 production. In a recent study, the authors identified prostaglandin E2 (PGE2) in the tumor as a key mediator of resistance to immunotherapies. PGE2 is coupled with a suppressive chemokine profile and the presence of high numbers of granulocytic myeloid-derived suppressor cells (MDSC) in the TME. Oncolytic VV expressing the prostaglandin-inactivating enzyme hydroxyprostaglandin dehydrogenase 15 or addition of the COX-2 inhibitor celecoxib mitigates local immunosuppression, leading to profound changes in the immune status of the TME. As such, these regimens could sponsor robust adaptive anti-tumor immunity within the TME, and sensitize tumors to interventional immunotherapies [138].

1. Combination with pharmaceutical drugs

Pharmaceutical drugs may be used to modulate the innate and/or adaptive immune environment in the TME for improved virotherapy. Studies have shown that combined OV with histone deacetylase inhibitors (HDIs) improved viral replication and therapeutic efficacy [139]. Two studies showed that HDIs could enhance viral replication and the spread of an oncolytic VV inside the tumor by dampening cellular IFN responses and augmenting virus-induced apoptosis [140, 141]. We explored this combination using a drug cocktail consisting of IFN-α, poly I:C, and a COX-2 inhibitor [142], which was previously shown to modulate the TME and expression of chemokines in vitro [143]. We showed that sequential treatment with an oncolytic VV and then the cocktail resulted in the upregulation of Th1-attracting chemokines and a reduction in levels of T*reg*-attracting chemokines (CCL22 and CXCL12), in association with enhanced trafficking of NK cells and tumor-specific CD8^+^ T cells into the TME. This combination led to pronounced anti-tumor activity and the extended long-term survival of mice bearing MC38 colon carcinomas. In another study, the authors combined the OV mpJX-594 with sunitinib (an oral, small-molecule, multi-targeted receptor tyrosine kinase inhibitor). This combined regimen worked through multiple mechanisms, with the virus targeting tumor blood vessels, spreading secondarily to tumor cells, and exerting tumor cell-killing mediation by CD8^+^ T cells, which were amplified by the immunomodulatory action of sunitinib [144]. These studies illustrate that concerted innate and adaptive antitumor immunity can be modulated via pharmaceutical agents and OVs in the TME to achieve improved therapeutic benefits.

2). Combination of OVs with immune checkpoint blockade

We have explored the efficacy of a combined regimens using oncolytic VV and anti-PD-L1 antibody in murine tumor models [98]. Our key hypothesis was that an OV would not only elicit an anti-tumor adaptive immune response, but also virus-induced inflammation leading to upregulation of PD-L1 expression by both tumor and stromal cells, making the environment conditionally-responsive to anti-PD-L1-based antagonism. Indeed, we observed virus-induced expression of PD-L1 in the tumor tissue, with the combination treatment regimen yielding superior anti-tumor efficacy and extended overall survival [98]. Furthermore, a new oncolytic VV-expressing superagonist IL-15 elicits potent antitumor immunity, and when combined with anti-PD-1 Ab as a therapy, treated animals displayed dramatic tumor regression in a murine colon carcinoma model [131]. Additionally, Fend et al. showed that intratumoral injection of an oncolytic VV significantly altered TME infiltration by lymphocytes and inflammatory myeloid cells, with notably more CD8^+^ TIL and fewer T*reg* cells post-treatment. When combined with an ICD inducer (oxaliplatin), anti-PD1, or anti-CTLA4, the anti-tumor efficacy of OV was enhanced in an MCA205 sarcoma model [121]. It is therefore fully expected that combinations of oncolytic VV and immune checkpoint blockade will soon be investigated in a clinical setting.

#### The EEV form of VV for immune evasion

One unique property of poxviruses is the existence of two infectious forms as described earlier: the IMV and EEV forms [32, 145]. IMV is known to infect cells less efficiently than EEV, which is surrounded by an additional, trans-Golgi network-derived membrane. When the IMV binds HeLa cells, it activates a signaling cascade that is regulated by multiple factors, including the GTPases rac1 and rhoA, ezrin, and phosphorylation of both tyrosine and protein kinase C [146]. Thus, the EEV membrane seems to have developed the capacity to silently enter cells. In 1996, Ichihashi observed that the EEV form of the virus escapes inactivation by neutralizing antibodies [147], and EEV has also been shown to resist complement due to the incorporation of host complement control proteins into its envelope [148]. The CEV/EEV outer membrane contains at least six viral proteins: A33, A34, A56, B5, F13, and K2 (for review, see Breiman and Smith, 2010) [149]. The glycoprotein A34R is required for infectivity of EEV [150]. Kirn et al. compared the oncolytic potential of low versus high EEV-producing strains of VV and showed that EEV-enhanced VV strains displayed improved spread within tumors after systemic delivery, resulting in significantly improved antitumor effects [109]. Our group has rationally designed A34R mutant (lysine-151 → glu) VV, resulting in greater production of EEV and improved therapeutic efficacy when applied in a peritoneal carcinomatosis model [30]. In the clinical setting, if an oncolytic VV is planned for repeated administration in the same patient, it will likely be preferred to deliver one that produces more of the EEV form after injection.

##### Different routes of delivery for oncolytic VVs

OVs are conventionally delivered to tumors via three routes: local (intratumoral), systemic (intravenous, i.v.), and locoregional (such as intraperitoneal [i.p.] delivery to tumor). Currently, the majority of preclinical studies have employed local OV injection. However, systemic delivery represents a major goal in the field of oncolytic virotherapy, as it would provide greater potential to effectively treat (potentially inaccessible) disseminated disease [151, 152]. Systemic delivery results in lower efficiency, a key reason for which is the rapid clearance of the viruses from the circulation before they reach their target sites. Tanabe and associates directly compared i.v. versus i.p. delivery of HSV-1 in a peritoneal tumor model in mice [153]. They concluded that i.p. administration of an oncolytic HSV-1 was associated with a far more restricted biodistribution, less toxicity, and greater efficacy against peritoneal metastases. Clinically, T-VEC has been only been approved to treat advanced melanoma via intralesional injection [154].

Theoretically, VV is ideally suited for systemic delivery, as it is partially resistant to complement and antibody-mediated neutralization in the blood. Due to its relatively large size, it is preferentially deposited in tumors where the abnormal neovasculature exhibits enhanced permeability allowing for enhanced viral entry into the TME. It can spread to distant tissues, which is crucial for its ability to treat systemic disease. Clinically, after i.v. administration of an oncolytic VV and dose-related delivery, viral replication, and transgene expression has been observed in metastatic tumor sites in humans [155]. In fact, the three oncolytic VVs in the clinical studies, namely Pex-Vec (Wyeth strain) [155, 156], vvDD (WR strain) [157], and GL-ONC1 (Lister strain) [158], have all been delivered intravenously to patients. As for which route is clinically superior, no randomized comparisons using oncolytic VV have yet been performed. In the absence of such information, we hypothesize that conclusions drawn from studies with other OVs will be applicable to oncolytic VVs.

##### Anti-vascular effects exerted by oncolytic VV

Pexa-Vec mediates the unexpected effect of disrupting the tumor-associated vasculature in both mice and humans [144, 159, 160]. Further studies have shown that Pexa-Vec can infect and replicate in tumor-associated vascular endothelial cells, with efficient replication and transgene expression in normal endothelial cells dependent on either VEGF or FGF-2 stimulation [160]. Bell and associates recently showed that this expanded tropism to tumor-associated endothelial cells is a consequence of VEGF-mediated suppression of the intrinsic anti-viral response. One key component in this anti-vascular mechanism of action is the induction of PRD1-BF1/Blimp1 expression [161]. In mice, this disruption led to massive tumor necrosis. In humans, intraveneous Pexa-Vec was used to treat advanced HCC, a hypervascular and VEGF-rich tumor type. Pexa-Vec treatment led to the disruption of tumor perfusion in as few as 5 days in both patients treated with this regimen. Based on this limited information, it seems that virus-induced anti-vascular activity may significantly contribute to therapeutic efficacy of this oncolytic VV. However, this supposition warrants verification as the massive necrosis induced by tumor-specific vascular collapse triggered by the VV may be highly-immunosuppressive, thus dampening the magnitude and/or durability of any resultant anti-tumor immunity.

### Clinical studies of VV as oncolytic virus

The development of Pexa-Vec showcases the advancement of oncolytic VVs from preclinical studies to clinical trialing. This virus was initially developed as a viral vector to express GM-CSF as a cancer vaccine and was then applied in patients with cutaneous melanoma in the late 1990s [63]. Only recently has this virus been rediscovered as an oncolytic virus and renamed JX-594, and later Pexa-Vec (by SilliJen Biotherapeutics) [125]. It has undergone testing in multiple phase I/II clinical trials in patients with HCC [155, 159, 162], where viral replication, GM-CSF secretion from infected cancer cells, and the induction of anti-tumor immune responses have been demonstrated. More importantly, the authors demonstrated that extended patient survival was significantly related to the dosage of the virus administered, with a median survival of 14.1 months compared to 6.7 months in the high- vs. low-dose treatment cohorts, respectively [162]. In a related study, Pexa-Vec has been shown to induce antibody-mediated, complement-dependent cancer cell lysis in cancer patients [163]. Currently, Pexa-Vec along with the TKI sorafenib are undergoing testing in a phase III PHOCUS global clinical study for patients with HCC.

Other strains of oncolytic VVs have been tested in early phase clinical trials. Lister strain-derived GL-ONC1 is safe in patients with locoregionally-advanced head/neck cancer undergoing standard chemoradiotherapy [158] or in patients with peritoneal carcinomatosis [164], and warrants further clinical studies. We have been studying the WR strain-derived OVs and have completed two phase I clinical trials of vvDD-CDSR in patients with advanced-stage solid cancers [157, 165]. In all of these clinical trials, no severe adverse effects have been reported. To date, we have observed some clinical responses, but only amongst melanoma patients, which may be related to its consensus as one of the most immunogenic forms of cancer [166, 167].

How do different types of cancer and different immune environments in the tumor, surrounding normal tissues, or organs impact OVs, especially oncolytic VV-mediated and immunotherapeutic OVs? These remain complicated issues that will need to be systematically evaluated for further optimization of OV-based therapeutic approaches. Based on numerous studies, a growing consensus is that there is a highly-positive correlation between cancer immunogenicity and efficacy of immunotherapy, including oncolytic immunotherapy. Melanoma is considered the most immunogenic type of cancer, and thus the most susceptible cancer to immunotherapy. For this reason, many clinical trials on cancer immunotherapy have been performed on melanoma, and the first oncolytic virus approved by the FDA is for patients with advanced melanoma. Some other types of cancers also display comparatively high immunogenicity, e.g., lung squamous cell carcinoma, bladder cancer, and colorectal cancer with MSI-H. These disease indications may also represent preferred targets for interventional oncolytic immunotherapy. The immune environment’s yin and yang roles in OV-mediated therapy is also an important consideration. On one hand, an immunosuppressive environment supports better viral replication, but this also dampens the induction of potent antitumor immunity. On the other hand, a more immunogenic environment promotes the premature clearance of OV, thus limiting its potential to activate and sustain therapeutic anti-tumor immunity.

## Conclusion and perspectives

Numerous studies have now shown that VVs and other poxviruses have limited therapeutic efficacy as cancer vaccines when used alone. In fact, this limited efficacy has been a general concern for the whole field of cancer vaccines in the past. Sub-optimal vaccine design and an immunosuppressive TME are the root causes for the inability of the immune system to mediate cancer eradication [168, 169]. As such, key areas for improvements in viral vector design must include the consideration of strategies to reverse the immunosuppressive TME. Therefore, one exciting area of research will be further improvements of poxvirus vectors. Table 1 lists over 20 candidate genes for genetic engineering to improve viral vector immunogenicity. However, it is important to note that even though increased immunogenicity of the virus will be advantageous to its use as a cancer vaccine, this represents a major disadvantage to an oncolytic virus, as the increased immune response to the virus will lead to premature clearance of the vector, thus reducing oncolytic potency. Thus, strategies need to be carefully planned when it comes to developing new recombinant poxvirus as a pure cancer vaccine vector or as an OV, which represents a special type of cancer vaccine.

It is worth pointing out again that VVs and other OVs could serve as excellent platforms for multimodal cancer therapeutics [8, 170]. They can be armed not only with genes for immune-stimulating, anti-angiogenic, and prodrug therapy, but also with reporter genes for imaging and serial therapeutic monitoring. VVs have been investigated in combination with chemo-, radio-, and other immunotherapeutic modalities. Some of these rational combinations have led to exciting therapeutic results in preclinical studies and warrant further clinical testing in human patients. Pexa-Vec combined with sorafenib in a global phase III trial for HCC showcases such a promising development.

A number of hurdles remain that limit the widespread use of oncolytic VV, just as is the case for other OVs [171, 172]. The first impediment is the limited efficiency of delivering OV to and propagating it throughout the entire tumor lesion, as well as, the ability to infect disseminated cancer cells over distance. In this regard, the EEV form of the virus may provide a tool to overcome such a hurdle, by evading clearance and permitting infect of distant tumor sites. A second hurdle involves the need to develop systemic antitumor immunity to impact disseminated disease, which typically evolves over time [173]. Third, recent evidence suggests that microbiota play an important role not only in the initiation, progression, and dissemination of a variety of cancers, but also in patient responsiveness to interventional immunotherapies, including immunogenic tumor cell death-inducing chemotherapies and immune checkpoint blockade. Currently, we have little knowledge for the potential role of microbiota in host responsiveness to VV or other OV-mediated cancer therapy. Finally, over 200 genes are encoded by the viral genome of VV, yet the functions of half of these genes remains unknown. Therefore, further understanding of the biology of the virus and viral gene functions is expected to improve our ability to manipulate these viruses to optimize their safety and efficacy when applied as cancer vaccines and/or oncolytic immunotherapies. In summary, despite these technical speed-bumps, the future of VV for use in cancer vaccines and oncolytic immunotherapies appears bright, especially when integrated in the setting of rational combination approaches that favor protective over regulatory immunity.
